# Distribution of acute symptoms and long COVID-19 and their association with anxiety and depression 2 years after infection

**DOI:** 10.3389/fpubh.2025.1687444

**Published:** 2026-01-08

**Authors:** Jingli Wen, Ling Yuan, Lin Zhang, Liang Li, Junyi Chen, Zhenjiang Zhang, Youde Yan

**Affiliations:** 1 Department of Infection, The Affiliated Suqian First People's Hospital of Nanjing Medical University, Suqian, China; 2 The Suqian Clinical College of Xuzhou Medical University, Suqian, China

**Keywords:** long COVID-19, anxiety, depression, mental health, medical staff

## Abstract

**Background:**

With the continuing impact of the COVID-19 outbreak, the mental health of infected patients is becoming a widespread concern. However, the relationship between acute-phase symptoms and long COVID-19 symptoms with anxiety and depression 2 years post-infection among healthcare workers remains unclear.

**Objective:**

The aim of this study was to investigate the relationship between acute phase symptoms and long COVID-19 symptoms of novel COVID-19 infection and anxiety and depression 2 years after infection.

**Methods:**

Using a retrospective cohort study and cross-sectional design, this study collected data on acute-phase symptoms, long COVID-19 symptoms, and their levels of anxiety and depression from 1,038 COVID-19 patients by questionnaire. We explored their relationship through logistic regression. We also used RCS curves to explore the nonlinear relationship between acute phase symptoms and long COVID-19 symptoms and anxiety and depression 2 years after infection.

**Results:**

The aim of this study was to investigate the prevalence of common symptoms of long COVID-19 in a sample of medical staff with COVID-19 infection. The study also aimed to explore the relationship between long COVID-19 symptoms and mental health status. The results showed that among patients who tested positive for COVID-19 by December 2022, approximately 34.0% exhibited overall long COVID-19 symptoms 2 years after infection. Decreased concentration and memory were the most common long COVID-19 symptoms, accounting for 12.5% of all COVID-19 patients. In this study, we also explored the relationship between acute-phase symptoms or long COVID-19 symptoms and anxiety and depression 2 years after infection. The findings showed that both acute phase symptoms as well as long COVID-19 symptoms were significantly associated with levels of anxiety and depression 2 years after infection. We also found a nonlinear relationship between the number of long COVID-19 symptoms and anxiety and depression.

**Conclusion:**

In summary, the positive correlation between the acute phase of COVID-19 infection and the impact of long COVID-19 symptoms on mental health suggests that focusing on the mental health of patients recovering from the epidemic is critical. Effective psychological interventions should be part of the comprehensive treatment of long COVID-19 to help patients improve their mental health while recovering physically.

## Introduction

Since late 2019, SARS-CoV-2 has rapidly expanded across the world, causing an unprecedented crisis in public health (1). COVID-19 infections usually last less than 4 weeks on average (2). In its acute phase, COVID-19 is characterized by a variety of symptoms, including cough, fever, and malaise, which can progress to severe respiratory distress and even result in death. The usual symptoms of acute COVID-19 include respiratory issues, fever, and neurological symptoms (3). The duration and severity of acute COVID-19 cases vary, with some individuals showing no symptoms while others need hospitalization and mechanical ventilation (2). As understanding of this novel virus has improved, researchers have also found that some infected individuals experience a range of long-lasting symptoms after recovery. This phenomenon is referred to as long COVID-19 (4). The definition of long COVID-19 adopted by the World Health Organization (WHO) is the persistence of symptoms or the emergence of new symptoms 3 months after SARS-CoV-2 infection, with symptoms persisting for at least 2 months and unexplained by other diagnoses (5). A variety of physical dysfunctions are connected to long COVID-19, demonstrating the multisystemic effects of the COVID-19 infection (6). Specifically, persistent COVID-related symptoms may affect the cardiorespiratory, neurologic, nasopharyngeal, gastrointestinal, and musculoskeletal systems (7, 8). Some symptoms, such as fatigue, lack of focus, and sleep issues, are also common in depression or anxiety.

Anxiety and depression are increasingly recognized as important mental health issues. The rise of anxiety and depression following COVID-19 infection has become a major public health concern globally. A growing body of research suggests that patients often face mental health challenges following COVID-19 infection, with the prevalence of anxiety and depression being significantly higher in patients who have recovered from COVID-19. Studies on the mental health impact of the COVID-19 pandemic have shown that healthcare workers have a higher prevalence of depression (18.7–29%) and anxiety symptoms (10.1–32%), and that both conditions persist beyond the peak of the COVID-19 pandemic (9, 10). This phenomenon is not only related to the physiological effects of the infection itself, but also to a combination of factors such as the social environment, individual psychological characteristics, and lifestyle. In the face of this challenge, it is particularly important to identify and intervene early in mental health problems following COVID-19 infection in order to provide patients with comprehensive support and treatment programs that can help them better adapt to life after their infection. Therefore, exploring the relationship between symptoms in the acute symptoms of COVID-19 infection or long COVID-19 symptoms and anxiety and depression approximately 2 years after infection is not only of theoretical importance but also has practical applications for the development of relevant interventions.

Preliminary evidence suggests that 2 years after COVID-19 infection, people with persistent COVID-19 symptoms have more severe mental health symptoms than people without persistent COVID-19 symptoms (11). A recent study explored the potential correlation between risk of long COVID-19 and mental health problems. Specifically, the study found that long COVID-19 was associated with increased depression and anxiety (12), which may be attributable in part to the persistent physical symptoms of long COVID-19 (13). In China, a small number of studies have focused on assessing the impact of long COVID-19 on the mental health of COVID-19 survivors. A study in China discovered that experiencing at least one long COVID-19 symptom raised the likelihood of developing depression or anxiety by 3.44 times (14). However, the research was restricted to patients who were hospitalized and recruited in 2020. No studies have been conducted in the medical workforce. Given the limitations of these studies, there is a need for further research on the impact of long COVID-19 on mental health using samples of medical staff.

The aim of this study was to explore the relationship between the acute symptoms of COVID-19 infection or long COVID-19 symptoms and anxiety and depression in medical staff 2 years after infection. We expect to reveal the relationship between the two through quantitative and qualitative research methods, with a view to providing a scientific basis for post-COVID-19 mental health interventions and support measures for healthcare professionals.

## Methods

### Participants

**Figure 1:**
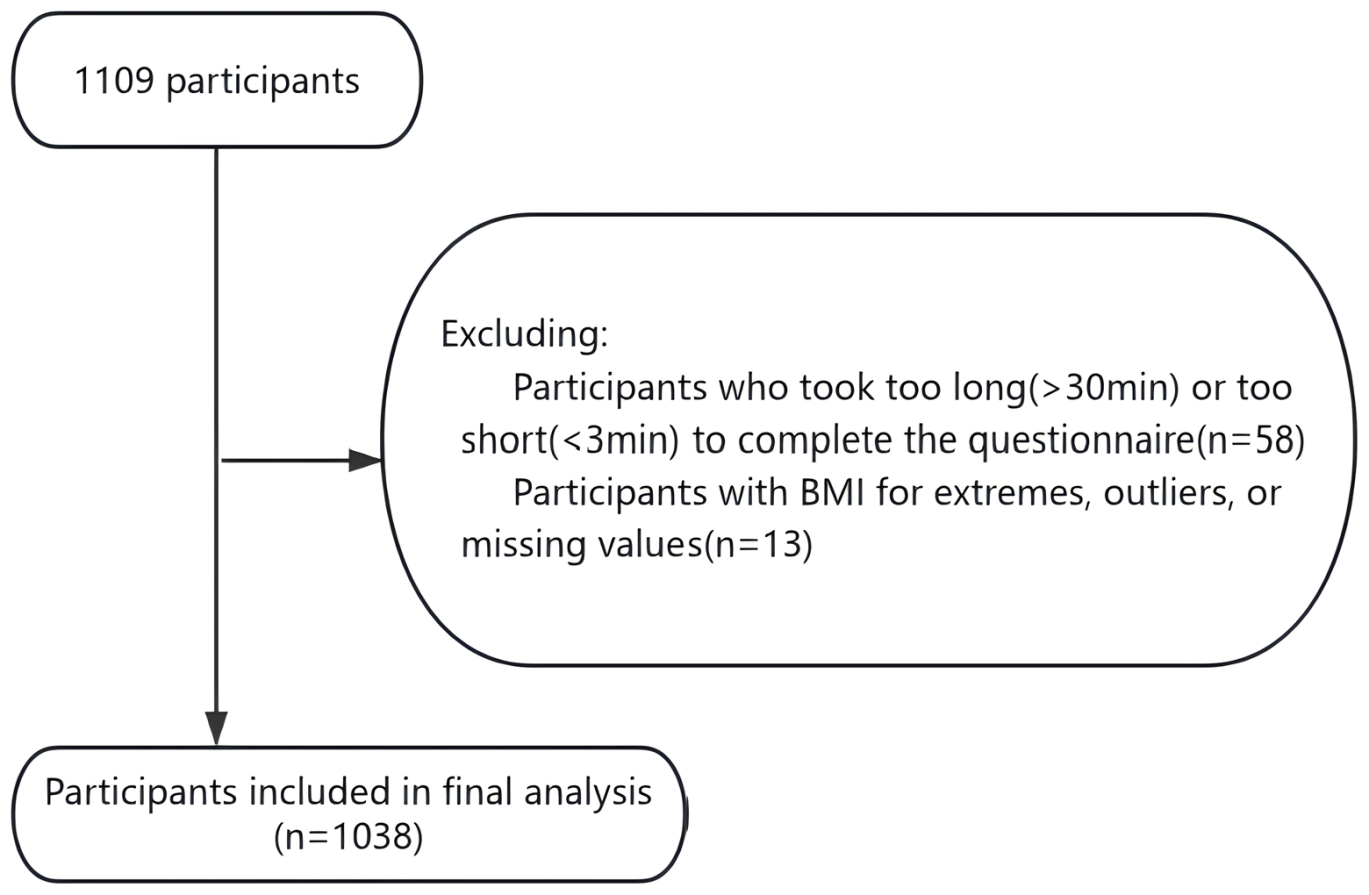
Flow chart of study population inclusion.

This was a retrospective cohort study based on a single-center medical staff survey of the entire medical staff of Suqian First People’s Hospital. The study population consisted of medical staff with confirmed COVID-19 infections between January 2020 and December 2022 in Suqian First People’s Hospital. Conducted in July and August 2024, the survey occurred approximately 20 months after the peak of COVID-19 infections in December 2022. This time point is more similar to 2 years after infection, and therefore, our text is mainly expressed as 2 years after infection. Participants who did not have a positive antigen test result, either from a hospital or self-test, in China by December 2022 were excluded. Participants indicated their consent by participating in the survey, which was stated in the promotional materials and on the survey home page. The study was approved by the Ethics Committee of Suqian First People’s Hospital (2024-SL-0046). A COVID-19 questionnaire was distributed to all hospital staff through Questionnaire Star, and 1,109 questionnaires were collected. After excluding those who answered the questionnaire for less than 3 min or more than 30 min and those with extreme values, 1,038 participants were included (Figure 1).

### Definitions of long COVID-19

Long COVID-19 was defined as persistent symptoms or new symptoms that persisted for at least 2 months after 3 months of SARS-CoV-2 infection and could not be explained by any other diagnosis (15). Symptoms of long COVID-19 were assessed through questionnaires, and individuals with one or more symptoms were considered to have long COVID-19.

### Measures

#### Demographic and health information

Demographic variables included sex, age in years, occupation, education, years of work, marital status, number of household members, and annual household income. Health information included body mass index (BMI), smoking, alcohol consumption, co-morbidity, and number of vaccinations.

#### Acute symptoms and long COVID-19

We used the COVID-19 Symptom Inventory to retrospectively collect acute symptoms, including fever, cough, fatigue, muscle aches and pains, and others, that occurred on days one, three, seven, and 30 during the participant’s infection (Supplementary Table 1). The scoring criteria are “yes” and “no.” We refer to the article published by Barak Mizrahi et al. to assess participants for long COVID-19 symptoms after COVID-19 infection (15), including fever, fatigue, loss of appetite, weight loss, and others, which we assessed at three time points of 180, 360, and 720 days, with retrospective collection in 180, and 360 days and cross-sectional survey in 720 days (Supplementary Table 2).

#### Anxiety and depression

The Patient Health Questionnaire-9 (PHQ-9 (16)), a 9-question instrument, was used to evaluate depression by assessing symptoms experienced in the last 2 weeks, showing strong criterion validity for diagnosing depression. Anxiety levels were evaluated using the Generalized Anxiety Disorder-7 (GAD-7 (17)), which includes seven anxiety symptoms assessed over the last 2 weeks. Participants were required to evaluate how often they experienced each symptom in the last 2 weeks for both the PHQ-9 and GAD-7, using a scale from 0 points for not at all to 3 points for nearly every day. Higher overall scores on the PHQ-9 (Cronbach’s *α* = 0.93) and GAD-7 (Cronbach’s α = 0.95) indicate greater depression and anxiety levels. Scores of 5 or above on these assessments suggest that participants are anxious or depressed.

### Statistical analysis

We categorized participants into two groups based on the presence or absence of long COVID-19 at 2 years after infection. Baseline characteristics are expressed as means (standard error [SE]) for continuous variables and as percentages for categorical variables. We used t-tests to compare demographic data for continuous variables, and differences in proportions were assessed using the χ^2^ test. Next, we used univariate logistic regression to explore the relationship between long COVID-19 symptoms at different times and anxiety and depression 2 years after infection; we then further examined the relationship using multivariate logistic regression to control for the effects of covariates. Finally, we analyzed the nonlinear relationship between the number of long COVID-19 symptoms at different periods and anxiety and depression scores using restricted cubic spline (RCS). All data cleaning and statistical analyses were performed in R version 4.0.3. For all statistical tests, *p* < 0 0.05 was considered significant.

## Results

### Participants characteristics

A total of 1,038 healthcare workers participated in our study who had previously reported a prior COVID-19 infection (Figure 1). Table 1 shows the demographic and health information of the participants and the comparison of the long COVID-19 population with the non-long COVID-19 population 2 years after infection. Overall, 86.8% of the participants were female (*n* = 901); the mean age was 31.06 years (SE = 2.67); 70.8% of the participants were nurses (*n* = 735); and 80.7% of the participants received three or four doses of the new coronary vaccine (*n* = 838). Of the 1,038 participants, 353 (353/1038, 8.89%) self-reported long COVID-19 symptoms 2 years after infection. The most common symptom was decreased attention and memory (12.5%), followed by fatigue (9.5%), hair loss (5.5%), headache (4.0%), and chest tightness (3.6%; Supplementary Table 2).

**Table 1:** Descriptive statistics of the study sample.

Variables	Total (*n* = 1,038)	Without long COVID of 2 years (*n* = 685)	With long COVID of 2 year (*n* = 353)	*p-*value
Gender				0.573
Male	137 (13.2)	87 (12.7)	50 (14.2)	
Female	901 (86.8)	598 (83.3)	303 (85.8)	
Age, year				<0.001
Mean ± SE	31.06 ± 2.67	30.44 ± 2.68	32.26 ± 2.63	
Occupation				0.128
Doctor	94 (9.1)	69 (10.1)	25 (7.1)	
Nurse	735 (70.8)	472 (68.9)	263 (74.5)	
Other	209 (20.1)	144 (21.0)	65 (18.4)	
Years of working, year				<0.001
≤ 5	362 (34.9)	267 (39.0)	95 (26.9)	
5–10	367 (35.4)	242 (35.3)	125 (35.4)	
10–20	246 (23.7)	138 (20.1)	108 (30.6)	
≥ 20	63 (6.1)	38 (5.5)	25 (7.1)	
Education				0.112
Less than undergraduate	190 (18.3)	136 (19.9)	32 (15.3)	
Undergraduate	783 (75.4)	503 (73.4)	155 (79.3)	
More than undergraduate	65 (6.3)	46 (6.7)	11 (5.4)	
Marital status				0.018
Married	364 (35.1)	258 (37.7)	106 (30.0)	
Unmarried	674 (64.9)	427 (62.3)	247 (70.0)	
Number of family members				0.155
1	57 (5.5)	31 (4.5)	26 (7.4)	
2–4	661 (63.7)	433 (64.7)	218 (61.8)	
≥ 5	320 (30.8)	211 (30.8)	109 (30.9)	
Annual household income, ten thousand				0.244
≤ 8	363 (35.0)	235 (34.3)	128 (36.3)	
8–15	415 (40.0)	286 (41.8)	129 (36.5)	
> 15	260 (25.0)	164 (23.9)	96 (27.2)	
BMI, kg/m^2^				0.040
≤ 24	740 (71.3)	503 (73.4)	237 (67.1)	
> 24	298 (28.7)	182 (26.6)	116 (32.9)	
Smoking status				0.200
No	1,013 (97.6)	672 (98.1)	341 (96.6)	
Yes	25 (2.4)	13 (1.9)	12 (3.4)	
Alcohol consumption				0.491
No	951 (91.6)	631 (92.1)	320 (90.7)	
Yes	87 (8.4)	54 (7.9)	33 (9.3)	
Co-morbidity				<0.001
No	946 (91.1)	643 (93.9)	303 (85.8)	
Yes	92 (8.9)	42 (6.1)	50 (14.2)	
Number of vaccinations				0.081
0–2	200 (19.3)	143 (20.9)	75 (16.1)	
3–4	838 (80.7)	542 (79.1)	296 (83.9)	

BMI, body mass index.

### Impact of acute phase symptoms on anxiety and depression

We first analyzed the effect of acute-phase symptoms of COVID-19 infection on anxiety and depression 2 years after the infection (Table 2). We found a significant positive correlation between the presence or absence of acute-phase symptoms—after adjusting for confounders—and levels of anxiety (3 day: OR: 2.45, CI [95%]: 1.33–4.76, *p* = 0.006; 7 day: OR: 2.21, CI [95%]: 1.57–3.15, *p* < 0.001; 30 day: OR: 1.94, CI (95%): 1.49–2.53, *p* < 0.001) and depression (3 day: OR: 3.62, CI [95%]: 1.87–7.60, *p* < 0.001; 7 day: OR: 2.55, CI [95%]: 1.80–3.65, *p* < 0.001; 30 day: OR: 2.19, CI (95%): 1.68–2.87, *p* < 0.001) 2 years after infection (except for acute-phase symptoms on the first day). Specifically, patients with acute-phase symptoms (e.g., fever, muscle aches, and malaise) reported significantly higher rates of anxiety and depression at 2 years.

**Table 2:** Relationship between symptom and anxiety or depression (scores as a categorical variable).

Symptom (Yes vs No)	Unadjusted model			Adjusted model		
	OR	CI (95%)	*p*	OR	CI (95%)	*p*
Anxiety						
1 day (98.4% vs. 1.6%)	1.44	0.53–4.25	0.487	1.21	0.42–3.76	0.724
3 day (94.7% vs. 5.3%)	2.38	1.33–4.51	0.005	2.45	1.33–4.76	0.006
7 day (81.5% vs. 18.5%)	2.22	1.60–3.12	<0.001	2.21	1.57–3.15	<0.001
30 day (59.1% vs. 40.9%)	2.07	1.61–2.67	<0.001	1.94	1.49–2.53	<0.001
180 day (50.7% vs. 49.3%)	3.36	2.61–4.35	<0.001	3.12	2.39–4.08	<0.001
360 day (38.2% vs. 61.8%)	3.28	2.53–4.27	<0.001	3.04	2.32–4.00	<0.001
720 day (34.0% vs. 66.0%)	2.93	2.25–3.83	<0.001	2.68	2.03–3.54	<0.001
Depression						
1 day (98.4% vs. 1.6%)	1.89	0.69–6.03	0.242	1.72	0.59–5.69	0.338
3 day (94.7% vs. 5.3%)	3.61	1.91–3.42	<0.001	3.62	1.87–7.60	<0.001
7 day (81.5% vs. 18.5%)	2.56	1.82–3.62	<0.001	2.55	1.80–3.65	<0.001
30 day (59.1% vs. 40.9%)	3.30	1.79–2.98	<0.001	2.19	1.68–2.87	<0.001
180 day (50.7% vs. 49.3%)	3.73	2.89–4.84	<0.001	3.50	2.68–4.60	<0.001
360 day (38.2% vs. 61.8%)	3.78	2.90–4.93	<0.001	3.17	2.24–4.52	<0.001
720 day (34.0% vs. 66.0%)	3.51	2.68–4.61	<0.001	3.26	2.47–4.34	<0.001

BMI, body mass index; CI, confidence interval; OR, Odds Ratio. Adjusted for gender, age, occupation, years of working, education, marital status, number of family members, annual of family income, BMI, smoking status, alcohol consumption, co-morbidity and number of vaccinations.

### Impact of long COVID-19 on anxiety and depression

To assess whether long COVID-19 affects patients’ mental status, we analyzed the effect of the presence or absence of long COVID-19 at three time points: 180 days, 260 days, and 720 days on anxiety and depression at 2 years (Table 2). Results showed that long COVID-19 symptoms such as fatigue and attention deficit that persisted after adjusting for confounders were also significantly correlated with levels of anxiety (180 day: OR: 3.12, CI [95%]: 2.39–4.08, *p* < 0.001; 360 day: OR: 3.04, CI [95%]: 2.32–4.00, *p* < 0.001; 720 day: OR: 2.68, CI (95%): 2.03–3.54, *p* < 0.001) and depression (180 day: OR: 3.50, CI [95%]: 2.68–4.60, *p* < 0.001; 360 day: OR: 3.17, CI [95%]: 2.24–4.52, *p* < 0.001; 720 day: OR: 3.26, CI (95%): 2.47–4.34, *p* < 0.001) 2 years after infection.

### Non-linear relationship between long COVID-19 and anxiety or depression

We further explored the nonlinear relationship between the number of long COVID-19 symptoms and anxiety and depression. We found a nonlinear relationship between the number of long COVID-19 symptoms and anxiety and depression at the 180-, 260-, and 720-day time nodes (*p* < 0.001). At the 180-day time point, when there were fewer than or equal to 3 long COVID-19 symptoms, it was positively associated with anxiety or depression 2 years after infection; when there were more than 3 long COVID-19 symptoms, it was no longer significantly positively associated with anxiety or depression. At the 360-day time point, it is positively correlated with anxiety or depression 2 years after infection when there are less than or equal to 2 symptoms; it is no longer significantly positively correlated with anxiety or depression when there are more than 2 symptoms. At the 720-day time point, it is positively associated with anxiety or depression 2 years after infection when the number of symptoms is less than or equal to 2; it is no longer significantly associated with anxiety or depression when the number of symptoms is greater than 2 (Supplementary Figures 1–6).

### Sensitivity analysis

We further analyzed the relationship between acute-phase COVID-19 symptoms and long COVID-19 with them by using anxiety and depression scores as continuous variables. We similarly found that all but acute-phase symptoms on the first day after infection were positively correlated with anxiety or depression scores (Table 3).

**Table 3:** Relationship between symptom and anxiety or depression (scores as a continuous variable).

Symptom	Unadjusted model			Adjusted model		
	*β*	CI (95%)	*p*	*β*	CI (95%)	*p*
Anxiety						
1 day (98.4% vs. 1.6%)	0.25	−2.07–2.57	0.834	−0.00	−2.32–2.32	1.000
3 day (94.7% vs. 5.3%)	1.90	0.63–3.18	0.003	1.79	0.51–3.06	0.006
7 day (81.5% vs. 18.5%)	1.98	1.25–2.71	<0.001	1.86	1.13–2.59	<0.001
30 day (59.1% vs. 40.9%)	1.84	1.27–2.41	<0.001	1.62	1.04–2.20	<0.001
180 day (50.7% vs. 49.3%)	2.81	2.26–3.36	<0.001	2.55	1.99–3.11	<0.001
360 day (38.2% vs. 61.8%)	3.02	2.46–3.58	<0.001	2.78	2.21–3.36	<0.001
720 day (34.0% vs. 66.0%)	3.00	2.42–3.58	<0.001	2.77	2.18–3.35	<0.001
Depression						
1 day (98.4% vs. 1.6%)	0.23	−2.19–2.65	0.852	0.08	−2.34–2.51	0.945
3 day (94.7% vs. 5.3%)	2.45	1.13–3.77	<0.001	2.31	0.99–3.64	0.001
7 day (81.5% vs. 18.5%)	2.30	1.55–3.06	<0.001	2.18	1.43–2.94	<0.001
30 day (59.1% vs. 40.9%)	2.17	1.58–2.77	<0.001	1.96	1.36–2.56	<0.001
180 day (50.7% vs. 49.3%)	3.15	2.59–3.71	<0.001	2.87	2.29–3.45	<0.001
360 day (38.2% vs. 61.8%)	3.48	2.90–4.05	<0.001	3.21	2.62–3.80	<0.001
720 day (34.0% vs. 66.0%)	3.49	2.90–4.08	<0.001	3.22	2.61–3.83	<0.001

BMI, body mass index; CI, confidence interval. Adjusted for gender, age, occupation, years of working, education, marital status, number of family members, annual of family income, BMI, smoking status, alcohol consumption, co-morbidity and number of vaccinations.

## Discussion

The aim of this study was to investigate the prevalence of common symptoms of long COVID-19 in a sample of medical staff with COVID-19 infection. The study also aimed to explore the relationship between long COVID-19 symptoms and mental health status. The results showed that among patients who tested positive for COVID-19 by December 2022, approximately 34.0% exhibited overall long COVID-19 symptoms 2 years after infection. Decreased concentration and memory were the most common long COVID-19 symptoms, accounting for 12.5% of all COVID-19 patients. In this study, we also explored the relationship between acute-phase symptoms or long COVID-19 symptoms and anxiety and depression 2 years after infection. The findings showed that both acute phase symptoms as well as long COVID-19 symptoms were significantly associated with levels of anxiety and depression 2 years after infection. We also found a nonlinear relationship between the number of long COVID-19 symptoms and anxiety and depression.

Several previous studies support our findings. In a meta-analysis of 12 studies covering 4,828 patients with COVID-19 infection, long COVID-19 was associated with poorer mental health (18). In our study, we further confirmed the effect of long COVID-19 at different time points on anxiety and depression 2 years after infection. After additional adjustments for confounders linked to COVID-19 infection, the impact on mental health was not significantly reduced. Our research agrees with past qualitative studies (19) which have found that those with long COVID-19 experience persistent physical or neurological symptoms that contribute to their mental health struggles. This intensification of physical symptoms could have further impacts, such as stopping involvement in regular coping strategies (20), such as exercise or breathing exercises, or decreasing socialization, potentially increasing feelings of loneliness and isolation (21). Depression and anxiety symptoms might worsen due to insufficient treatment options and uncertainty regarding the condition’s duration (19).

For the relationship between long COVID-19 and mental health, changes in daily life as a result of the epidemic, such as economic instability, social distancing, and wearing masks, challenged the psychological needs that people had been meeting for the past few years (22–24). In addition, for patients who develop symptoms of long COVID-19, they may experience other symptoms related to brain function, resulting in poorer mental health compared to patients who recover well from COVID-19 infection (25). Thus, the burden of physical illness increases the prevalence of mental illness.

The results highlight the importance of providing mental health assistance to individuals severely impacted by COVID-19, particularly those with long COVID-19 symptoms. In 2020, China’s National Health and Wellness Commission released COVID-19 Rehabilitation Program for Discharged Patients (26), which recommends respiratory exercises, physical workouts, mental health support, and activities to regain daily living skills to tackle respiratory, physical, and psychological issues like coughing, tiredness, and anxiety that might continue. So far, treatment approaches for long COVID-19 at specialized or outpatient clinics have mainly aimed at alleviating symptoms, with both Western and Chinese medicine emphasizing physical rehabilitation (27, 28). Overall, mental health services, especially for patients showing symptoms of Long New Crown, are insufficient (29). There is a shortage of mental health rehabilitation services, and telemedicine is not widely used, mainly because facilities are limited (30). Due to limited resources, it is crucial to develop and validate prevention programs using a mHealth approach for early detection, intervention, treatment, and management of long COVID-19 symptoms (31, 32).

In the present study, although we explored the relationship between acute-phase symptoms of COVID-19 infection and long COVID-19 symptoms with anxiety and depression 2 years after infection, there are some limitations that may affect the generalization and interpretation of the results. First, sample size and selection may affect the extrapolation of results. The participants in this study were medical staff in a single hospital and were predominantly nurses, which may not be fully representative of the entire population of medical staff COVID-19 infected patients, especially since patients in different regions, cultures, or socio-economic backgrounds may show different results. This selection bias may prevent us from fully recognizing the impact of acute phase symptoms and long COVID-19 symptoms on anxiety and depression in healthcare workers. Second, there were limitations in how the data were collected. This study relied on self-report questionnaires, which may have been influenced by participants’ subjective feelings and cognitive biases. Many patients may have been influenced by social expectations when reporting their psychological state or failed to accurately express it because of their own emotional state. In addition, some participants may have failed to clearly remember their specific symptoms and their severity during the acute phase, which may affect the accuracy of the study results. Third, the study used a cross-sectional and retrospective cohort study design, which meant that we were only able to analyze the data at a specific point in time and were unable to establish causality. Although we observed correlations between acute phase symptoms and long COVID-19 symptoms with anxiety and depression, it was not possible to determine how these symptoms impacted mental health or, conversely, how psychological states impacted symptom presentation. In addition, this study failed to consider potential confounders. For example, factors such as prior mental health history, social support networks, and life events may play an important role in this relationship. Future studies should try to control for these confounding variables in order to more clearly interpret the effects of acute phase symptoms and long COVID-19 symptoms on anxiety and depression. Finally, the definition and categorization of long COVID-19 symptoms has not been agreed upon across studies, which may lead to difficulties in comparison across studies. Therefore, standardized symptom assessment tools are needed to advance research in this area.

## Conclusion

In summary, our study found that acute-phase symptoms and long COVID-19 symptoms had a significant negative impact on mental health. This suggests that it is crucial to pay attention to the mental health issues of healthcare workers during the recovery phase after an outbreak. Effective psychological interventions should be part of the comprehensive treatment of long COVID-19 to help patients improve their mental health while recovering physically. Future research needs to continue to explore the deeper mechanisms of long COVID-19’s effect on mental health, as well as more individualized treatment protocols, with a view to providing a more comprehensive treatment strategy.

## Data Availability

The raw data supporting the conclusions of this article will be made available by the authors, without undue reservation.
