# Supplementary material for: Distribution of acute symptoms and long COVID-19 and their association with anxiety and depression 2 years after infection
Source: Front Public Health. 2026 Jan 8;13:1687444. doi: 10.3389/fpubh.2025.1687444 (PMC12823984; doi:10.3389/fpubh.2025.1687444)
Supplement: Supplementary file 1 [file Supplementary_file_1.docx]

**Supplemental Online Content**

**eFigure 1.** Nonlinear association between long COVID-19 symptoms of 180 days after infection and anxiety.

**eFigure 2.** Nonlinear association between long COVID-19 symptoms of 360 days after infection and anxiety.

**eFigure 3.** Nonlinear association between long COVID-19 symptoms of 720 days after infection and anxiety.

**eFigure 4.** Nonlinear association between long COVID-19 symptoms of 180 days after infection and depression.

**eFigure 5.** Nonlinear association between long COVID-19 symptoms of 360 days after infection and depression.

**eFigure 6.** Nonlinear association between long COVID-19 symptoms of 720 days after infection and depression.

**eTable 1.** The frequency (%) of acute phase symptoms of COVID-19 in the sample.

**eTable 2.** The frequency (%) of long COVID-19 in the sample.

**
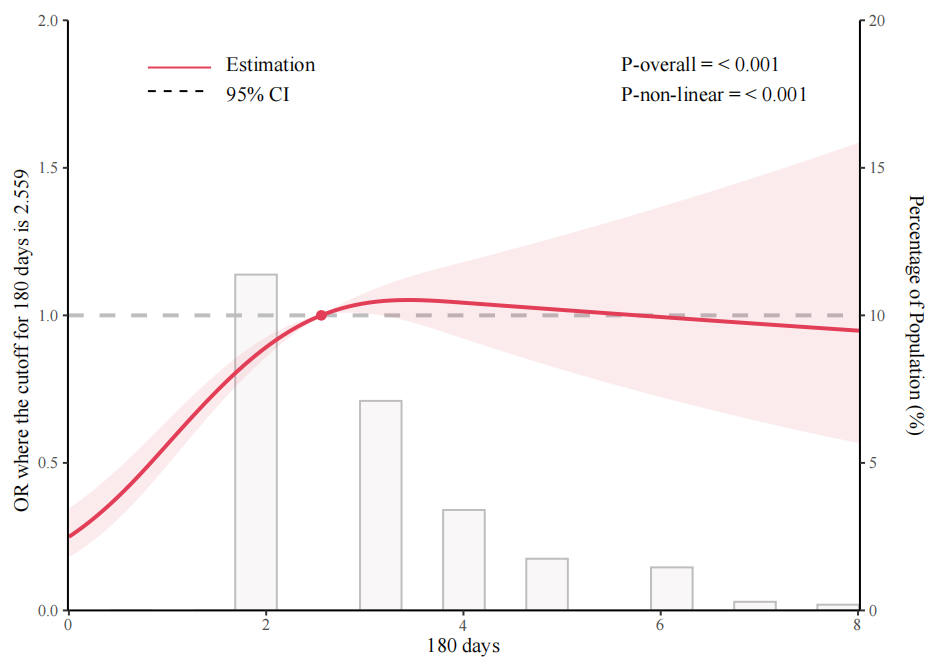
**

**eFigure 1.** Nonlinear association between long COVID-19 symptoms of 180 days after infection and anxiety.


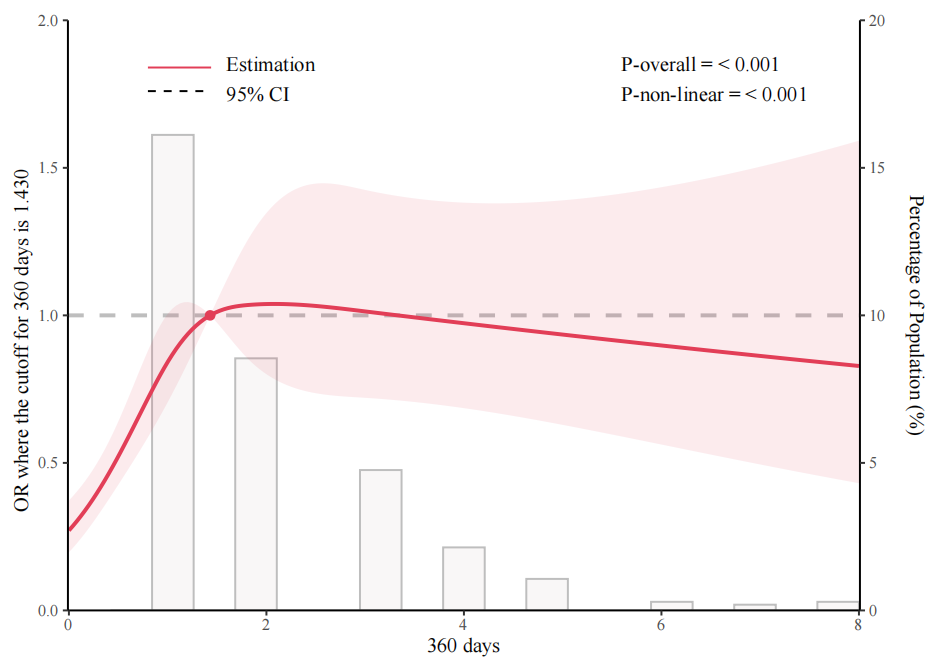


**eFigure 2.** Nonlinear association between long COVID-19 symptoms of 360 days after infection and anxiety.


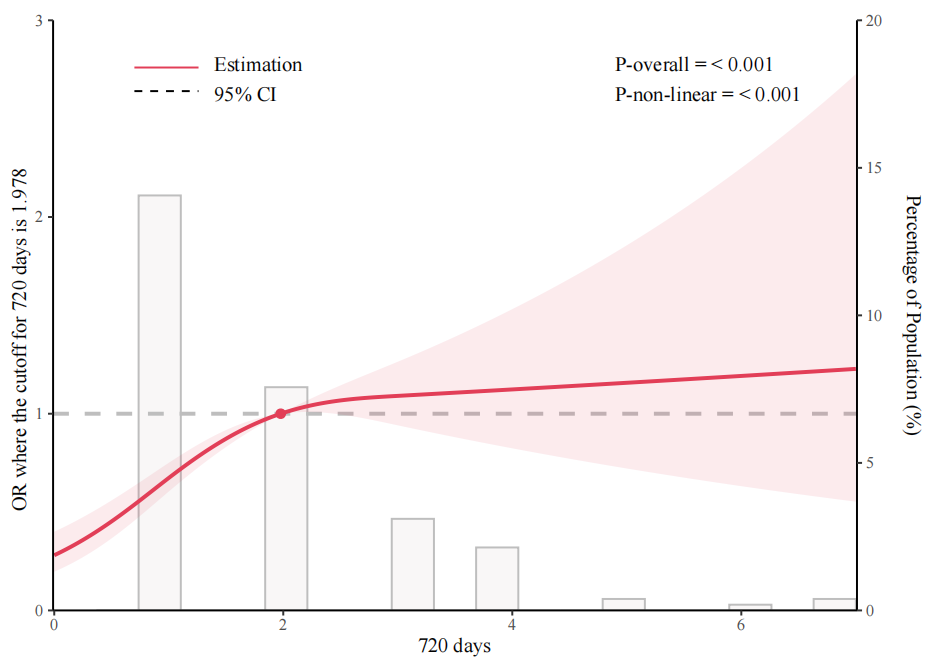


**eFigure 3.** Nonlinear association between long COVID-19 symptoms of 720 days after infection and anxiety.


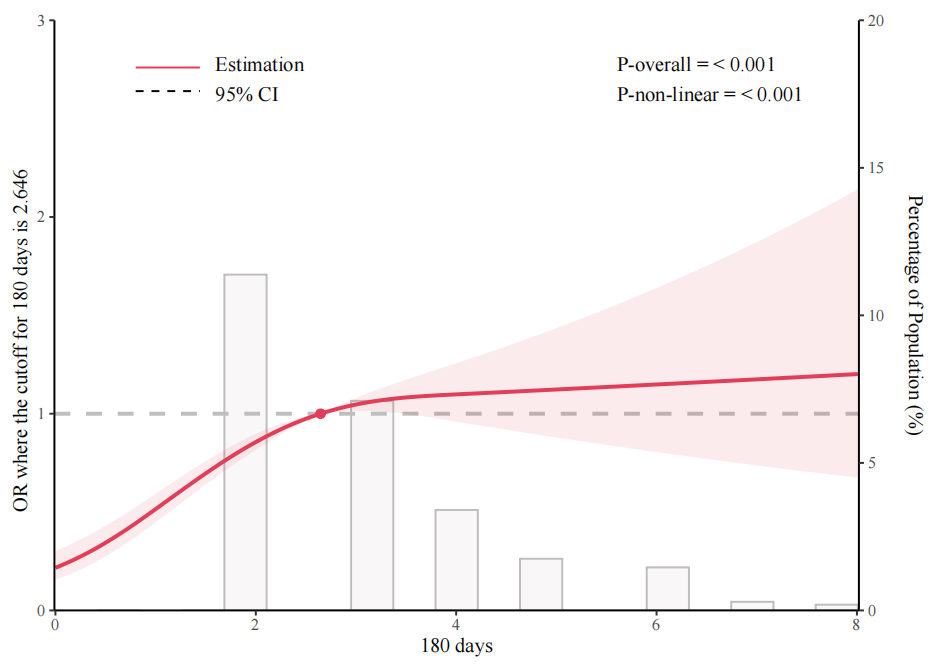


**eFigure 4.** Nonlinear association between long COVID-19 symptoms of 180 days after infection and depression.


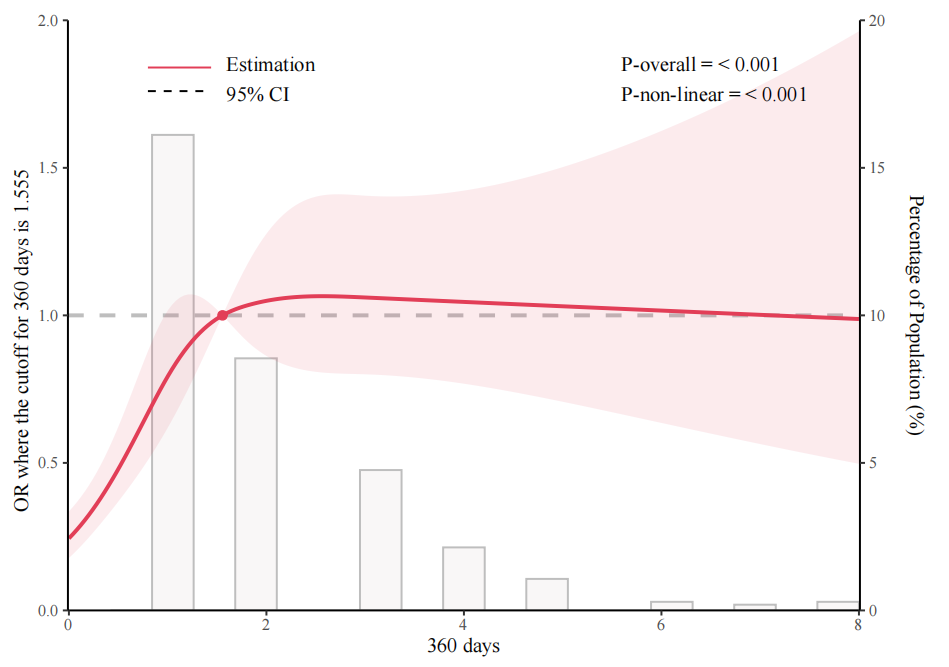


**eFigure 5.** Nonlinear association between long COVID-19 symptoms of 360 days after infection and depression.


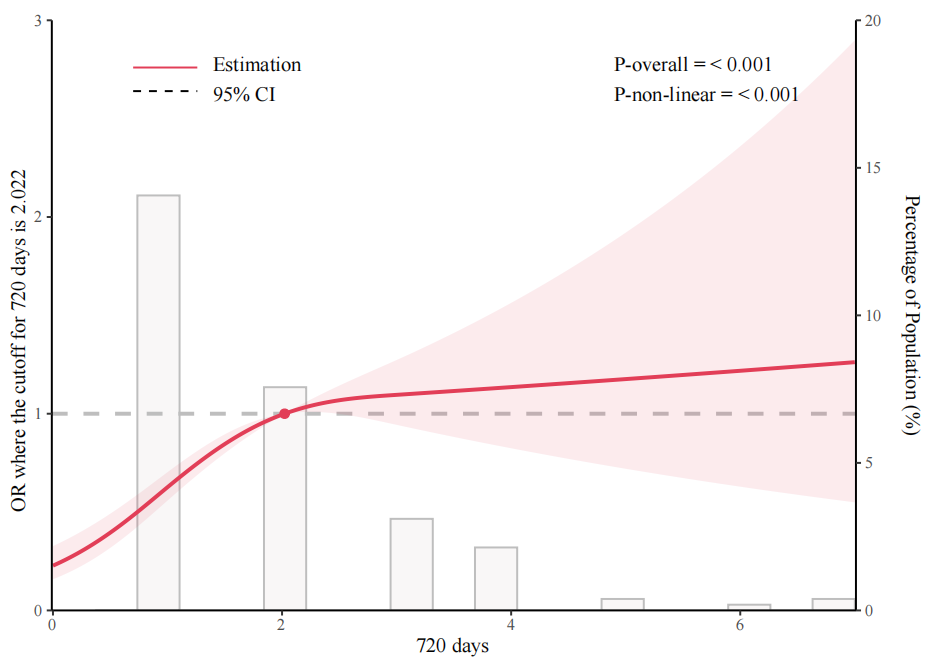


**eFigure 6.** Nonlinear association between long COVID-19 symptoms of 720 days after infection and depression.

**eTable 1.** The frequency (%) of acute phase symptoms of COVID-19 in the sample.

|  | **Frequency (%)** | | | |
| --- | --- | --- | --- | --- |
| **Acute symptoms** | **1 day** | **3 day** | **7 day** | **30 day** |
| Fever | 89.4 | 43.9 | 11.7 | 4.3 |
| Sore throat | 75.9 | 58.1 | 31.2 | 11.6 |
| Hoarseness | 36.6 | 34.3 | 19.9 | 7.9 |
| sneezing | 44.5 | 37.4 | 22.0 | 6.8 |
| cough | 32.1 | 34.8 | 30.2 | 19.3 |
| expectoration | 26.8 | 28.9 | 19.8 | 10.0 |
| fatigue | 57.8 | 47.0 | 36.7 | 26.3 |
| Muscle soreness | 62.3 | 40.9 | 22.5 | 9.5 |
| Loss of smell and taste | 21.8 | 21.6 | 14.7 | 8.1 |
| diarrhea | 3.6 | 3.3 | 1.3 | 0.7 |
| Vomit | 4.6 | 3.2 | 1.1 | 0.5 |
| Conjunctivitis | 1.8 | 1.1 | 0.8 | 0.6 |
| Chest tightness | 14.2 | 10.3 | 10.1 | 9.2 |
| Any acute COVID-19 symptoms | 98.4 | 94.7 | 81.5 | 59.1 |

**eTable 2.** The frequency (%) of long COVID-19 in the sample.

|  | **Frequency (%)** | | |
| --- | --- | --- | --- |
| **Long COVID-19** | **180 day** | **360 day** | **720 day** |
| Fever | 3.4 | 2.6 | 2.4 |
| Fatigue | 17.9 | 11.4 | 9.5 |
| Loss of appetite | 6.5 | 3.6 | 2.2 |
| Weight loss | 2.9 | 1.6 | 1.3 |
| Chest pain | 4.1 | 3.5 | 2.7 |
| Muscle soreness | 4.8 | 3.2 | 2.2 |
| headache | 6.0 | 4.6 | 4.0 |
| Sore throat | 4.3 | 2.0 | 1.9 |
| Chest tightness | 9.5 | 3.3 | 3.6 |
| cough | 7.7 | 3.8 | 2.7 |
| Nausea | 0.4 | 0.3 | 0.2 |
| diarrhea | 0.4 | 0.4 | 0.3 |
| tinnitus | 1.8 | 1.7 | 1.4 |
| Joint pain | 2.7 | 2.0 | 2.0 |
| Night sweats | 3.2 | 1.9 | 1.0 |
| stomach ache | 0.2 | 0.2 | 0.1 |
| Blood in the stool | 0.3 | 0.1 | 0.1 |
| rash | 1.0 | 0.6 | 0.4 |
| Visual impairment | 0.5 | 0.8 | 0.5 |
| Menstrual abnormalities | 4.2 | 2.6 | 2.0 |
| Smell or taste disturbances | 3.3 | 2.0 | 0.6 |
| Hair loss | 7.4 | 6.6 | 5.5 |
| Decreased concentration and memory | 19.3 | 14.7 | 12.5 |
| Any long COVID-19 symptoms | 50.7 | 38.2 | 34.0 |
